# LncCDH5-3:3 Regulates Apoptosis, Proliferation, and Aggressiveness in Human Lung Cancer Cells

**DOI:** 10.3390/cells11030378

**Published:** 2022-01-23

**Authors:** Konrad Kwaśniak, Justyna Czarnik-Kwaśniak, Khrystyna Malysheva, Katarzyna Pogoda, Olexandr Korchynskyi, Paweł Rybojad, Bożenna Karczmarek-Borowska, Jacek Tabarkiewicz

**Affiliations:** 1 Centre for Innovative Research in Medical and Natural Sciences, Medical College of Rzeszów University, 35-310 Rzeszow, Poland; justynaa.czarnik@gmail.com (J.C.-K.); khrystyna.malysheva@gmail.com (K.M.); kateann89@gmail.com (K.P.); olexkor@hotmail.com (O.K.); jacek.tabarkiewicz@gmail.com (J.T.); 2 Department of Immunology, Institute of Medical Sciences, College for Medical Sciences, University of Rzeszow, 2a mjr Kopisto St., 35-959 Rzeszow, Poland; 3 Department of Biotechnology and Radiology, S. Gzhytskyi National University of Veterinary Medicine and Biotechnologies, 50 Pekarska St., 79010 Lviv, Ukraine; 4 Department of Molecular Immunology, Palladin Institute of Biochemistry of the National Academy of Sciences of Ukraine, 6 Leontovicha St., 01161 Kyiv, Ukraine; 5 Department of Thoracic Surgery, Medical University of Lublin, 8 Jaczewskiego St., 20-097 Lublin, Poland; rybojad@wp.pl (P.R.); 6 Medyk Medical Centre, 53 al. Tadeusza Rejtana, 35-055 Rzeszow, Poland; bkb8@tlen.pl (B.K.-B.)

**Keywords:** lncCDH5-3:3, non-small cell lung cancer, apoptosis, proliferation, migration, tumor cell aggressiveness

## Abstract

(1) Lung cancer (both small cell and non-small cell) is the leading cause of new deaths associated with cancers globally in men and women. Long noncoding RNAs (lncRNAs) are associated with tumorigenesis in different types of tumors, including lung cancer. Herein, we discuss: (1) An examination of the expression profile of lncCDH5-3:3 in non-small cell lung cancer (NSCLC), and an evaluation of its functional role in lung cancer development and progression using in vitro models; (2) A quantitative real-time polymerase chain reaction assay that confirms lncCDH5-3:3 expression in tumor samples resected from 20 NSCLC patients, and that shows its statistically higher expression levels at stage III NSCLC, compared to stages I and II. Moreover, knockout (KO) and overexpression, as well as molecular and biochemical techniques, were used to investigate the biological functions of lncCDH5-3:3 in NSCLC cells, with a focus on the cells’ proliferation and migration; (3) The finding that lncCDH5-3:3 silencing promotes apoptosis and probably regulates the cell cycle and E-cadherin expression in adenocarcinoma cell lines. In comparison, lncCDH5-3:3 overexpression increases the expression levels of proliferation and epithelial-to-mesenchymal transition markers, such as EpCAM, Akt, and ERK1/2; however, at the same time, it also stimulates the expression of E-cadherin, which conversely inhibits the mobility capabilities of lung cancer cells; (4) The results of this study, which provide important insights into the role of lncRNAs in lung cancer. Our study shows that lncCDH5-3:3 affects important features of lung cancer cells, such as their viability and motility. The results support the idea that lncCDH5-3:3 is probably involved in the oncogenesis of NSCLC through the regulation of apoptosis and tumor cell metastasis formation.

## 1. Introduction

Lung cancer is one of the most frequent neoplasms in the world, and it is a serious medical and socioeconomic problem, especially in highly developed countries. Non-small cell lung cancer (NSCLC) is the leading cause of cancer-related deaths worldwide, in both men and women, with increased morbidity in young adults [1]. The molecular basis of lung cancer, especially NSCLC, is complex, heterogeneous, and is still not fully understood. It is well known that long noncoding RNAs (lncRNAs) can regulate the proliferation, migration, and invasion capacities of cancer cells [2]. Moreover, the disease severity, metastases occurrence, and, therefore, the survival of patients can be affected by lncRNAs [3].

LncRNAs belong to the class of noncoding RNAs that are longer than 200 nucleotides and that do not possess protein-coding abilities [4,5]. Most importantly, from a medical point of view, these molecules are tissue-specific, and such tissue specificity is even higher than in mRNA’s [3,6]. LncRNAs play important regulatory roles in many biological processes. In particular, lncRNAs regulate gene and protein expression at the epigenetic, transcriptional, post-transcriptional, translational, and post-translational levels by diverse classes of mechanisms [4,5,7]. Because of the important role of these molecules in regulating alternative splicing, cell differentiation, and cell cycle regulation, lncRNAs are also involved in regulating the occurrences of many diseases [5,8]. In cancer cells, especially, lncRNAs could be dysregulated qualitatively and quantitatively. This means that differences in the copy numbers or gains, as well as losses in the lncRNAs expression, may lead to many irregularities in the cells, and may, thus, result in disease development [9].

Studies have shown that lncRNAs regulate several signaling pathways, mediated by TGF-β, EGFR, or Wnt/β-catenin. Moreover, lncRNAs regulate the NSCLC tumor response to chemotherapy [8,10]. Despite all of the abovementioned roles of lncRNAs, they are still poorly understood, and more investigations are needed to broaden knowledge about the role of these molecules in malignancies.

The main objective of this study was to find lncRNA, which is transcribed before the *CDH1* gene. An in silico analysis of lncRNAs from Chr.16 led to the obtainment of very stable lncCDH5-3:3. The results presented in the LncBook database show the methylation of lncCDH5-3:3. The average values of the promoter and the lncCDH5-3:3 sequence methylation were higher in adenocarcinoma and squamous cell carcinoma, compared to healthy donors [11]. Moreover, this transcript is entirely within a long terminal repeat (LTR) sequence, which is less than a 10% divergence from the human endogenous retrovirus subfamily H (HERVH) consensus sequence. The active elements of HERVH can cause many diseases, including cancer [12]. Moreover, transposable elements can also produce lncRNAs that are highly expressed in human cells [13]. These results may suggest the significant role of lncCDH5-3:3 in tumorigenesis. Additionally, the NONCODE database presents information about lncCDH5-3:3 under the Transcript ID number: NON-HSAT142976.2. The information curated in this database also includes the expression profiles of lncRNAs determined by RNA sequencing [14].

In our research, we studied the role of an approximately 600-bp-long sense intergenic ncRNA, named “lncCDH5-3:3”, in the malignancy of NSCLC. LncCDH5-3:3 is localized on Chr.16 (68737225-68835548), upstream of the *CDH1* gene, which encodes E-cadherin. This protein inhibits the migration capacity of cancer cells and counteracts the epithelial-to-mesenchymal transition (EMT) [15]. The main objective of this study was to examine whether this lncRNA influences E-cadherin, and also EpCAM and β-catenin, because it can regulate the expressions of the CDH1 and EpCAM genes. We also focused on the tumor-growth-related molecular mechanisms involved in lung cancer development and progression. The proteins that are studied in this experiment, and that are arranged for proliferation, are ERK1/2 and Akt. Moreover, we also investigated the roles of Oct-4 and Nanog, which are responsible for the self-renewal of cancer cells.

We hypothesize that lncCDH5-3:3 controls CDH1 and, as a consequence, EPCAM expression, which encodes EpCAM, one more protein involved in the regulation of EMT, thereby exerting an important influence on the progression of lung adenocarcinoma [16]. To confirm our hypothesis, we performed a study of the lncCDH5-3:3 expression levels in NSCLC patients and their correlations with CDH1 and EPCAM expressions. We also performed an in vitro investigation on the effect of lncCDH5-3:3 silencing and overexpression on the cell cycle, proliferation, apoptosis, and expression of CDH1 and EPCAM in the adenocarcinoma and squamous cell carcinoma cell lines. Additionally, we studied the effects of the changes in the expressions of EpCAM and E-cadherin, and also those of other important proteins (Akt, ERK1/2, Nanog, Oct-4, and β-catenin) involved in the EMT, and the proliferation and/or migration of cancer cells [17,18].

## 2. Materials and Methods

### 2.1. Patients and Preparation of Tumor Tissue Samples

Tumor tissue samples were taken from 20 patients with NSCLC during surgeries performed at the Department of Thoracic Surgery Independent Public Teaching Hospital, No. 4 (Medical University of Lublin, Lublin, Poland). The mean patient age was 69.3 ± 7.84. The diagnoses of NSCLC were histologically confirmed, and the disease stages were categorized as I–III (adenocarcinoma: I-*n* = 3, II-*n* = 4, III-*n* = 3; squamous cell carcinoma I-*n* = 3, II-*n* = 3, III-*n* = 4), according to the 7th edition of the lung cancer tumor-node-metastasis (TNM) classification and staging system. The Supplementary Information in Table S1 summarizes the patient staging. After the surgeries, the tumor tissue samples were immersed in a 0.9% sodium chloride solution (Fresenius Kabi Polska Sp. z o.o., Warsaw, Poland), immediately cut into small pieces, and homogenized using a gentleMACS™ Dissociator (Miltenyi Biotec GmbH; Bergisch Gladbach, Germany). Thereafter, the suspensions were passed through 70-µm mesh strainers (Falcon^®^, Corning Life Science, Tewksbury, MA, USA), and the separate cells were washed twice with phosphate-buffered saline (PBS), purchased from GE Life Sciences/PAA Laboratories (Chalfont, UK). The procedure was performed in accordance with the Declaration of Helsinki and was approved by the Bioethics Committees of the Medical University of Lublin (No. KE-0254/235/2015) and the University of Rzeszów (No. 1/01/2015). Written informed consent was obtained from all of the patients enrolled in the study.

### 2.2. In Silico Prediction of RNA Secondary Structure

An in silico analysis of the possible lncCDH5-3:3 secondary structure was performed using the Vienna RNA Website software from Vienna University (http://rna.tbi.univie.ac.at/cgi-bin/RNAWebSuite/RNAfold.cgi, accessed on 17 April 2021). The predicted secondary structure of lncCDH5-3:3 was generated on the basis of its primary sequence.

### 2.3. Cell Culture

The A549 (human lung adenocarcinoma; ATCC^®^ CCL-185™), H1975 (human lung adenocarcinoma; ATCC^®^ CRL-5908™), and H1703 (squamous cell carcinoma; ATCC^®^ CRL-5889™) cell lines were purchased from the American Type Culture Collection (ATCC; Manassas, VA, USA). The A549 cells were cultured in Dulbecco’s Modified Eagle’s Medium/Nutrient Mixture, F-12 Ham (Sigma Aldrich/Merck; Steinheim, Germany), in a 1:1 ratio, whereas the H1975 and H1703 cells lines were cultured in an RPMI-1640 medium (Sigma Aldrich/Merck; Steinheim, Germany). All the media types used for the cell cultures were supplemented with 10% fetal bovine serum (FBS), purchased from EurX (Gdańsk, Poland), and 1% penicillin/streptomycin, purchased from Biowest (Nuaillé, France). The studied cell lines were grown at 37 °C, in a humidified atmosphere, with 5% CO_2_. The culture medium was refreshed every 2–3 days. Once the cells reached 70–80% of confluence, they were detached with a 0.25% accutase solution (Corning; New York, NY, USA). Before subcultivation, the cells were washed with PBS without magnesium and calcium (Biowest; Nuaillé, France). The cell morphology was monitored using a Primovert inverted microscope (ZEISS; Oberkochen, Germany). The lung cancer cells were seeded at a density of 4x10^4^ cells/well, in flat-bottom 24-well culture plates, in all the experiments (VWR International; Radnor, PA, USA). The cells were allowed to attach and grow for 48 h before treatment.

All of the studied cell lines were checked for mycoplasma contamination before use in the experiments. For this purpose, a Mycoplasma PCR Detection Kit was used (ScienCell Research Laboratories; Carlsbad, CA, USA).

### 2.4. LncCDH5-3:3 Knockout and Overexpression Procedure

The knockout of the lncCDH5-3:3 expression was performed using the CRISPR-Cas9 system. The gRNAs shown in Table 1 were used in the lung cancer cell lines to knockout lncCDH5-3:3. Because of the off-target effect, three gRNAs were designed. Efficient cleavage within the tested lncRNA was achieved using gRNA_1F and gRNA_3R (Figure S1). The gRNAs were cloned into pSpCas9(BB)-2A-Puro (PX459) V2.0 plasmid (Addgene plasmid #62988; http://n2t.net/addgene:62988, accessed on 15 June 2021 [19]), which was a gift from Prof. Feng Zhang (Massachusetts Institute of Technology, Cambridge, MA, USA). The cloning procedure was performed using annealing oligos, T7 DNA ligase (New England Biolabs; Ipswich, MA, USA), and a BbsI restriction enzyme (Thermo Fisher Scientific; Waltham, MA, USA), according to the protocol of Prof. Feng Zhang Labs (Massachusetts Institute of Technology, USA) [20]. The empty plasmid, px459 v2.0, was used as a control. To detect the efficiency of the lncCDH5-3:3 knockout 24, 48, and 72 h post-transient transfection in lung cancer cells (Figure S2), TiTaq polymerase (EurX, Gdańsk, Poland)) and the following primers were used: forward 5′-AGGAGGCTTTGGATTGGGAA-3′, and reverse 5′-TTACCCAATCTGCTCCCGAC-3′. In order to confirm the results obtained using the CRISPR-Cas9 method, we also used pcDNA3.1 plasmid. The full-length lncCDH5-3:3 sequence was annealed using the following primers: forward 5′-AGCCTAATAAGGGAACTGGGCA-3′, and reverse 5′-CTGTGAGACAAACCCCAGCCACAT-3′. Furthermore, the obtained lncCDH5-3:3 cDNA was recloned into a pcDNA3.1 vector, using T4 DNA ligase (Thermo Fisher Scientific; Waltham, MA, USA) and restriction enzymes: KpnI and XhoI (both enzymes from Thermo Fisher Scientific; Waltham, MA, USA). Empty pcDNA3.1 plasmid was used as a control. Both the gRNAs and the lncCDH5-3:3 sequence were validated using Sanger sequencing.

### 2.5. Transfection of Cell Line

The cells of the A549, H1975, and H1703 lines were transfected with px459 v2.0 and the appropriate gNRAs (for lncCDH5-3:3 knockout), and pcDNA3.1 plasmid, which contains the whole lncCDH5-3:3 sequence (for lncCDH5-3:3 overexpression), or control plasmids: empty px459 v2.0 and empty pcDNA3.1, respectively. The transfection was carried out using Lipofectamine™ (Invitrogen; Waltham, MA, USA), according to the manufacturer’s instructions. Cells with stable overexpressions of lncCDH5-3:3 were selected using G418 (1000 µg/mL, Cayman Chemical, Ann Arbor, MI, USA), 48 h after transfection. Because of the problems with obtaining viable cells after the lncCDH5-3:3 knockout, cells were not selected by puromycin.

### 2.6. RNA Isolation and qRT-PCR

Total RNA from the lung cancer cell lines (A549, H1975, and H1703) and the samples of NSCLC patients was isolated using a DNA/RNA Extracol kit (EurX; Gdańsk, Poland). The TURBO DNA-free kit, purchased from Invitrogen (Waltham, MA, USA), was used to remove the DNA from the samples. Thereafter, the RNA was transcribed into the cDNA using a High-Capacity RNA-to-cDNA Kit (Invitrogen; Waltham, MA, USA). For this purpose, a thermocycler, purchased from Syngen Biotech (Taiwan), was used. To detect the lncCDH5-3:3 expression levels in patients and the A549, H1975, H1703 cells, real-time PCR (Cobas 4800 System/Roche; Basel, Switzerland) was performed. For this purpose, the ExiLERATE LNA™ qPCR SYBR^®^ Green Master Mix (Exiqon A/S/Qiagene; Hilden, Germany), and the specific primers for amplification (forward 5′-CAGATGGGACGCGGCTTA-3′, and reverse 5′-ACACGAGAACTTCCAAACGC-3′), were used. The basal expression levels of lncCDH5-3:3 in the investigated cell lines are presented in Figure S3a. The changes in the EpCAM (Hs00158980_m1, Applied Biosystems; Waltham, MA, USA) and E-cadherin (Hs01023894_m1, Applied Biosystems; Waltham, MA, USA) mRNA levels in the A549, H1975, H1703 cells and the control cells after the lncCDH5-3:3 knockout were estimated by TaqMan Real-time PCR assays (Applied Biosystems; Waltham, MA, USA). Human hypoxanthine phosphoribosyltransferase 1, or HPRT1, was used as an endogenous control (Hs02800695_m1, Applied Biosystems; Waltham, MA, USA). In order to evaluate its expression level, the following primers were used: forward 5′-GCTATAAATTCTTTGCTGACCTGCTG-3′, and reverse 5′-AATTACTTTTATGTCCCCTGTTGACTGG-3′). The relative mRNA expressions of lncCDH5-3:3, EpCAM, and E-cadherin were calculated with the comparative threshold cycle (Ct) (2^−ΔCt^) method. To evaluate the overexpression level of lncCDH5-3:3, semiquantitative RT-PCR was performed. To perform this method, forward 5′-AGCCTAATAAGGGAACTGGGCA-3′ and reverse 5′-CTGTGAGACAAACCCCAGCCACAT-3′ primers were used. Equal cDNA loading of each lane was evaluated on the 1% agarose gel, with HPRT1 annealed using forward 5′-GAAGAGCTATTGTAATGACC-3′ and reverse 5′-GCGACCTTGACCATCTTTG-3′ primers. The signal capture was performed with a G-box machine (Syngene, Cambridge, UK). The densitometric intensities of the bands on the 1% agarose gel were assessed using ImageJ^®^ software (version 4.8) (Figure S3b).

### 2.7. Apoptosis Estimation

#### 2.7.1. Caspase-3/7 Activity Assay

The evaluation of the caspase-3/7 activity, which is a hallmark of apoptosis, was performed using the MuseTM caspase-3/7 assay kit (Merck Millipore; Burlington, MA, USA), according to the manufacturer’s protocol. Briefly, the A549, H1975, and H1703 cell lines were transfected with px459 v2.0 plasmid and the appropriate gRNAs. After 24, 48, and 72 h post transient transfection, the cells were washed, collected, and incubated with the 1X MuseTM Assay Buffer BA. Afterwards, incubation with MuseTM Caspase-3/7 Reagent for 30 min in a 37 °C incubator (Panasonic; Gunma, Japan), with 5% CO_2_, was performed. Next, MuseTM Caspase 7-AAD (dead cell marker) solution was added to the cells, which was followed by 5 min of incubation at room temperature. The percentages of the living cells (caspase-3/7 (−) and 7-AAD (−)), the dead cells (caspase-3/7 (−) and 7-AAD (+)), the apoptotic cells exhibiting caspase-3/7 activity (caspase-3/7 (+) and 7-AAD (−)), and late apoptotic/dead (caspase-3/7 (+) and 7-AAD (+)) cells were assessed using the Muse^®^ Cell Analyzer (Merck Millipore; Burlington, MA, USA). The estimation of the apoptosis was performed with the use of cells from three biological repeats. Figure S4 shows an example of the dot plots and the gating strategy used in this study.

#### 2.7.2. Annexin V Staining

The apoptosis was estimated using the Annexin V Dead Cell Kit (Merck Millipore; Burlington, MA, USA), according to the manufacturer’s protocol. Briefly, the A549, H1975, and H1703 cell lines were transfected with px459 v2.0 plasmid and the appropriate gRNAs After 24, 48, and 72 h of transfection, the cells were washed, collected, and incubated with MuseTM Annexin V and Dead Cell Reagent 7-AAD (dead cell marker) reagents for 20 min, in the dark and at room temperature. The percentages of living, dead, early, and late apoptotic cells were measured by a Muse^®^ Cell Analyzer (Merck Millipore; Burlington, MA, USA). The estimation of the apoptosis was performed with the use of cells from three biological repeats. Figure S5 shows an example of the dot plots and the gating strategy used in this study.

### 2.8. Cell Cycle Assay

The percentages of the cells in the G0/G1, S, and G2/M phases of the cell cycle were measured by a Muse^®^ Cell Analyzer (Merck Millipore; Burlington, MA, USA), using the Muse^®^ Cell Cycle Kit (Merck Millipore; Burlington, MA, USA), according to the manufacturer’s recommendations. In short, the A549 H1975 and H1703 cells were transfected with px459 v2.0 plasmid and the appropriate gRNAs after 24, 48, and 72 of transfection, and they were washed, collected, and incubated with Muse™ Cell Cycle Reagent for 30 min before analysis. Additionally, to confirm the apoptosis induction after the lncCDH5-3:3 knockout and the results gained from the test based on the caspases-3/7 activity or the cell membrane integrity, we have also gated the SubG1 population. The cell cycle assay was performed with the use of cells from three biological repeats. Figure S6 shows an example of the dot plots and the gating strategy used in this study.

### 2.9. Western Blotting

The A549, H1975, and H1703 cell lines were split, at density, in 4x10^4^ cells/wells in flat-bottom 24-well culture plates. The next day, the cells were transfected with an empty pcDNA3.1 plasmid, which contained the whole lncCDH5-3:3 sequence. After the next 48 h, a total cellular protein extraction was performed using RIPA buffer (Thermo Fisher Scientific; Waltham, MA, USA), supplemented with PMSF Protease Inhibitor (Thermo Fisher Scientific; Waltham, MA, USA). The protein concentrations in the extracts were measured using the Pierce BCA Protein Assay Kit (Thermo Fisher Scientific; Waltham, MA, USA), and 20 µg of the total cellular protein per well was subjected to electrophoresis in 12% SDS/PAGE gel. The resolved proteins were transferred onto polyvinylidene difluoride membranes (PVDF) that were purchased from Merck Millipore (Burlington, MA, USA). The wet transfer was carried out overnight, at 30 V, in the presence of a Towbin buffer (25 mM Tris, 192 mM glycine, and 20% methanol (*v*/*v*), with a pH of 8.3). Thereafter, the blots were incubated in a blocking solution, consisting of 3–5% BSA in a TBST buffer (20 mM Tris, 150 mM NaCl, and 0.1% Tween 20, with a pH of 7.5), for 1 h at room temperature. The target protein detection blots were incubated with primary antibodies overnight at 4 °C. We used mouse monoclonal antibodies against: EpCAM (R&D Systems; Minneapolis, MN, USA), at a dilution of 1:500; E-cadherin (Novus Biologicals; Littleton, CO, USA), at a dilution of 1:500; Oct-4 (R&D Systems; Minneapolis, MN, USA), at a dilution of 1:500; Nanog (Invitrogen; Carlsbad, CA, USA), at a dilution of 1:500; Akt (R&D Systems; Minneapolis, MN, USA), at a dilution of 1:500; phosphorylated-Akt (R&D Systems; Minneapolis, MN, USA), at a dilution of 1:500; ERK1/2 (R&D Systems; Minneapolis, MN, USA), at a dilution of 1:500; phosphorylated ERK1/2 (R&D Systems; Minneapolis, MN, USA), at a dilution of 1:500; and phosphorylated β-catenin (Merck; Darmstadt, Germany), at a dilution of 1:1000. Peroxidase-labeled secondary antibodies, antimouse IgG HRP Affinity Purified PAb, purchased from R&D Systems (Minneapolis, MN, USA), were added, at a dilution of 1:1000, to the TBST buffer containing 5% BSA, and were incubated with the blots for 1 h at room temperature. An ECL Western Blotting Kit (Thermo Fisher Scientific; Waltham, MA, USA) was used for chemiluminescent detection, according to the manufacturer’s instructions. The signal capture was performed with a G-box machine (Syngene). The densitometric intensities of the bands on the blots were assessed using ImageJ^®^ software (version 4.8). The equal protein loading of each lane was evaluated by the immunoblotting of the same membrane with anti-β-actin monoclonal antibodies at dilution (1:1000) (R&D Systems; Minneapolis, MN, USA).

### 2.10. Statistical Analysis

All of the experiments were performed in three biological replicates. The data were analyzed using Statistica 13.1PL software (StatSoft, Kraków, Poland) and GraphPad Prism 8.0.1 software (GraphPad Software, San Diego, CA, USA). The distribution of the variables was assessed with the use of the Shapiro–Wilk test. The nonparametric test was applied because of the non-Gaussian distribution of the variables. The medians, as descriptive statistics, and the minimum–maximum values, as measures of the dispersion, were used. For the testing differences between the groups of patients in different stages of disease, we used the Krukal–Wallis test, followed by the Dunn’s multiple comparisons test for post hoc analysis. The Wilcoxon test was applied to compare the two dependent variables, while for three or more dependent variables, the Friedman ANOVA and Dunn’s multiple comparisons post hoc tests were used. The level of the statistical significance of the test was *p* < 0.05. The cut-off points for the grouping of individual patients, according to the high (*CDH1* > 0.13; *EpCAM* > 1.6; *lncCDH* > 3.3) and low (*CDH1* < 0.13; EpCAM < 1.6; *lncCDH5-3:3* < 3.3) expressions of lncCDH5 3:3, EpCAM, and CDH1, were determined using the median of the expression values. The expression level values that were higher than the median of the mentioned genes and lncRNA were marked as “high”, while expressions lower than the median were marked as “low” levels of expression. The analysis was performed with the use of Statistica 13.1PL (StatSoft, Poland) and GraphPad Prism 8.0.1 software (GraphPad Software, USA).

## 3. Results

### 3.1. LncCDH5-3:3 Expression in NSCLC Patients and Its Correlations with CDH1 and EPCAM Gene Expression

**Figure 1:**
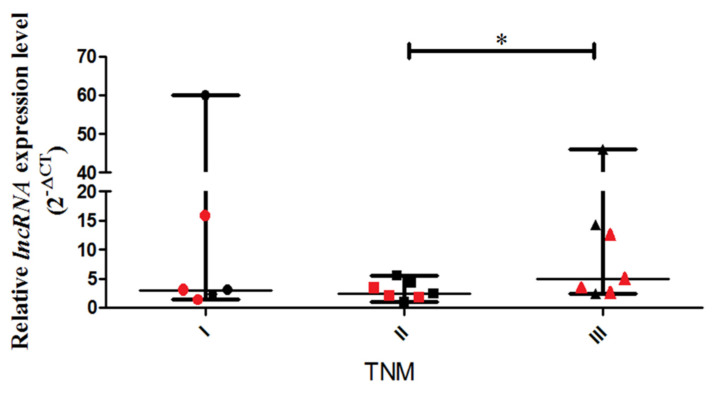
Relative expressions (2^−ΔCt^) of lncCDH5-3:3 in tumor samples from patients with NSCLC in stages I, II, and III of the disease. The diagnosis of NSCLC was histologically confirmed, and the disease stages were categorized from I–III (adenocarcinoma: I-*n* = 3, II-*n* = 4, III-*n* = 3; squamous cell carcinoma: I-*n* = 3, II-*n* = 3, III-*n* = 4) The data are presented as medians and minimum and maximum values. The *p*-values were calculated on the basis of the Friedman ANOVA and post hoc Dunn’s comparison tests. All statistically significant differences are marked: * *p* < 0.05. In this figure, the minimum values are marked as 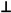; the maximum values as 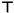; the median as **—**; and 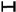 represents the *p*-value from the post hoc test. The circles, squares, and triangles filled with the red color represent squamous cell carcinoma samples, while the black circles, squares, and triangles mark the adenocarcinoma samples.

A real-time PCR assay was performed to detect the relative expression levels of lncCDH5-3:3 in the tumor tissue samples obtained from NSCLC patients with disease stages categorized as I, II, and III. The study shows a statistically higher (*p* < 0.05) expression level of lncCDH5-3:3 in stage III patients, compared to stage II patients (Figure 1).

**Figure 2:**
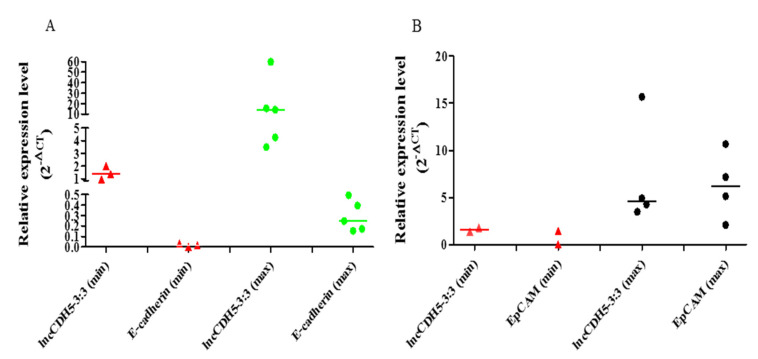
Correlations in relative expressions (2^−ΔCt^) of lncCDH5-3:3 and CDH1 (**A**) or EPCAM (**B**) genes in tumor samples from patients with NSCLC. Low and high expressions marked as “min” and “max”, respectively: for CDH1, the min > 0.13 and the max < 0.13; for EPCAM, the min > 1.6 and the max < 1.6; for IncCDH5-3:3, the min > 3.3 and the max > 3.3. The medians in these figures, (**A**,**B**), are marked as **—**, and the expression values of the lncCDH5-3:3, E-cadherin, or EpCAM patient samples are marked as red triangles and green or black circles, respectively.

The medians of the lncCDH5-3:3 relative expressions in the groups of patients, according to the stages of the disease, were stage I: 3.01; stage II: 2.48, and stage III: 13.41. The fact that many patients with stage III lung cancer have advanced disease, such as T factor 3, suggests a trend of slow metastases, but vigorous local growth. On the other hand, no statistically significant differences were found between the tumor types and the lncCDH5-3:3 expression levels. The median of the lncCDH5-3:3 relative expression for squamous cell carcinoma was 3.01, and for adenocarcinoma, it was 4.04. In order to determine the correlations between the lncCDH5-3:3 expression and the CDH1 or EPCAM expressions in patients with NSCLC, the medians of the relative expressions of these genes and lncCDH5-3:3 were calculated. It was shown that the medians of the CDH1 and EPCAM relative expressions were 0.13 and 1.6, respectively, while for lncCDH5-3:3, it was 3.3. Then, the samples were divided into low- and high-expression groups, on the basis of the lncCDH5-3:3, CDH1, and EPCAM relative expressions. Samples with relative expressions below 0.13 and 1.6 (CDH1 and EPCAM, respectively), and below 3.3 for lncCDH5-3:3, were defined as “low expression”, while samples with relative expressions higher than these values were defined as “high expression” (Figure 2A,B). It was found that the expression of lncCDH5-3:3 correlates with the CDH1 or EPCAM expressions. Particularly, as is shown in Figure 2A, in the group of NSCLC patients (*n* = 7), the same expressions were observed for CDH1 and lncCDH5-3:3 (*n* = 3 for low expression, and *n* = 4 for high expression). The same expressions were also detected in the group of six patients with NSCLC (*n* = 2 for low expression, and *n* = 4 for high expression) for EPCAM and lncCDH5-3: 3 (Figure 2B).

### 3.2. Analysis of LncCDH5-3:3 RNA Structure

We analyzed the primary sequence of lncCDH5-3:3 RNA and found five AUG codons at the positions of 132, 169, 255, 423, and 577 (Figure S1a). Of these, the first one overlaps with an optimal Kozak consensus motif (GUU-AUG-G) at position 132 because of guanines in both the −3 and +4 positions. A very similar optimal Kozak consensus motif (GUG-AUG-G) is also presented at the third AUG (position 255). On the other hand, in the lncCDH5-3:3 sequence, we also found stop codons: UAA at the positions of 5, 8, 31, 35, 49, 91,165, 197, and 306; UAG codons at the positions of 75, 100, 181, 261, 436, and 495; and UGA codons at the positions of 64, 156, 193, 225, 386, 391, and 601 (Figure S1a). The primary sequence of lncCDH5-3:3 RNA was further used for the prediction of its secondary structure, with Vienna RNA Website software from Vienna University. As is shown in Figure S1b, a predicted optimal secondary structure consists of multiple stem–loop structures. Moreover, the Gibbs’s free energy (ΔG) for the optimal lncCDH5-3:3 RNA stem–loop structure is −241.38 kcal/mol. Thus, the lncCDH5-3:3 transcript is supposed to be very stable.

The obtained results show that lncCDH5-3:3 is a stable molecule, of which the expressions vary, depending on the stage and type of NSCLC, and that may regulate the expression of EMT-associated genes, such as CDH1 and EPCAM. Thus, we also performed a functional study of CRISPR/Cas9-mediated lncCDH5-3:3 knockout (KO), and overexpression outcomes on the CDH1 and EPCAM expression, cell cycle, proliferation, and apoptosis in lung cancer cells. The investigation was carried out on the A549, H1975, and H1703 cell lines, which are extensively used in NSCLC studies as in vitro models [13,14].

### 3.3. Effects of LncCDH5-3:3 Silencing on CDH1 and EPCAM Expressions, as well as on Cell Cycle and Apoptosis in Lung Cancer Cell Lines

We determined the influence of lncCDH5-3:3 silencing on the expression of CDH1 and EPCAM, which are involved in the EMT. Moreover, EpCAM also regulates the proliferation of cancer cells [21]. For this purpose, lncCDH5-3:3 knockout in NSCLC cell lines was performed using the CRISPR-Cas9 system. To detect the expression levels of CDH1, EPCAM, and lncCDH5-3:3 after the knockout, real-time PCR was performed.

**Figure 3:**
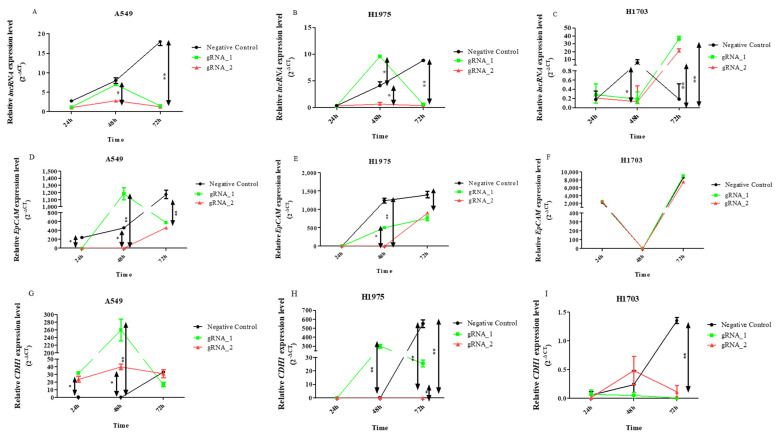
Relative expressions (2^−ΔCt^) of lncCDH5-3:3 (**A**–**C**), EPCAM (**D**–**F**), and CDH1 (**G**–**I**), in A549, H1975, and H1703 cells, after lncCDH5-3:3 silencing. Expressions were evaluated at 24, 48, and 72 h post transient transfection, using RNA isolated from cultured cells. Data are presented as medians and minimum and maximum values. The *p*-values were calculated on the basis of the Friedman ANOVA and Dunn’s comparison post hoc tests. All statistically significant differences are marked: * *p* < 0.05 and ** *p* < 0.01. In this figure, the maximum values are marked as 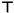; the minimum values are marked as 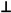; the medians are represented by circles, squares, and triangles; 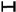 represents the *p*-value from the post hoc test.

Increased expression levels of lncCDH5-3:3 in the transfected cells were observed (Figure 3A–C). This can be explained by the cellular response to stress caused by transfection. The phenomenon is best demonstrated in the control cells of the A549 and H1975 lines, which were transfected with empty plasmid (Figure 3A,B), or in cells in which the lncCDH5-3:3 knockout failed (Figure 3A,B). It was also shown that, in the lung adenocarcinoma cell lines (A549 and H1975), lncCDH5-3:3 regulates the expression of CDH1 (Figure 3G,H). After KO, the lncCDH5-3:3 expression level of CDH1, in the case of A549, remains at a high constant level; however, in H1975, it decreases (Figure 3G,H). Additionally, 48 h after KO, adenocarcinoma and squamous carcinoma (H1703) cells have increased expressions of EpCAM (Figure 3I). This result indicates that EpCAM may be an important protein for NSCLC cells.

Thus, the change in the lncCDH5-3:3 expression created a stress condition, which, in turn, led to lncCDH5-3:3 overexpression (Figure 3A–C). Moreover, the obtained results demonstrate the likely important role of lncCDH5-3:3 in lung cancer cell proliferation and/or apoptosis. To study the effect of the lncCDH5-3:3 knockout on apoptosis and the cell cycle in NSCLC cell lines, a caspase-3/7 activity assay, Annexin V staining, and a cell cycle assay were performed.

**Figure 4:**
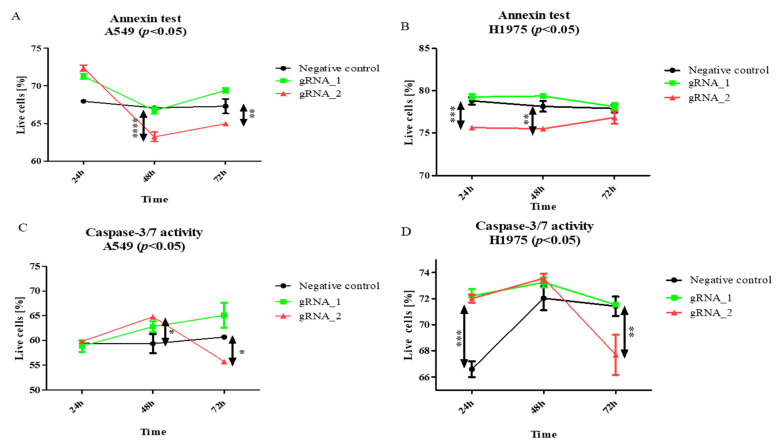
The effects of lncCDH5-3:3 silencing on the living A549 or H1975 cells (percentage of cultured cells). The apoptosis estimation was carried out by Annexin V staining (**A**,**B**) and a caspase-3/7 activity assay (**C**,**D**). Analysis was performed at 24, 48, and 72 h post transient transfection. Data are presented as medians and minimum (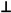) and maximum (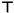) values from three biological replicates. The *p*-values were calculated on the basis of the Friedman ANOVA and Dunn’s comparative post hoc tests. Statistically significant results are marked: * *p* < 0.05, ** *p* < 0.01, *** *p* < 0.001, and **** *p* < 0.0001. In this figure, the medians are represented as circles, squares, and triangles, and 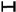 represents the *p*-value from the post hoc test.

The results of the Annexin V staining (Figure 4A) show that lncCDH5-3:3 silencing significantly lowered the percentage of viable A549 cells, in comparison to the control cells. This effect was observed 48 h (*p* < 0.0001) and 72 h (*p* < 0.01) after transfection. However, in the H1975 cell line, statistically significant decreases in the percentages of viable cells were observed after 24 h (*p* < 0.001) and 48 h (*p* < 0.01), compared to the control (Figure 4B). On the other hand, no decrease in the percentage of viable cells was determined in the squamous cell carcinoma cell line (data not shown). Moreover, as is shown in Figure 4C,D, the lncCDH5-3:3 knockout significantly increased the number of adenocarcinoma cells (*p* < 0.05 for the A549 line, and *p* < 0.01 for the H1975 line), showing caspase-3/7 activity 48 h post transient transfection, which indicates a decrease in the percentage of viable cells.

**Figure 5:**
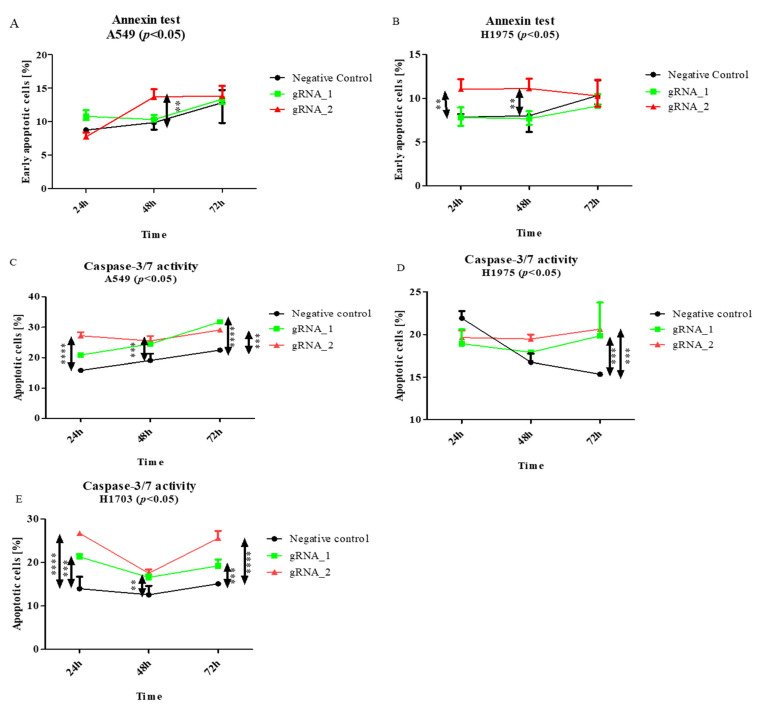
The effects of lncCDH5-3:3 silencing on the percentage of cultured cells of early apoptosis (**A**,**B**), and apoptotic (**C**–**E**) A549, H1975, and H1703 cells. The apoptosis estimation was carried out by Annexin V staining and a caspase-3/7 activity assay. Analysis was performed at 24, 48, and 72 h post transient transfection. Data are presented as medians and minimum (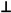) and maximum (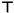) values from three biological replicates. The *p*-values were calculated on the basis of the Friedman ANOVA and Dunn’s comparative post hoc tests. Statistically significant results are marked: ** *p* < 0.01, *** *p* < 0.005, and **** *p* < 0.0005. In this figure, the medians are represented by circles, squares, and triangles, and 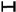 represents the *p*-value from the post hoc test.

As is shown in Figure 5A,B, significant changes (*p* < 0.01) were observed in the percentages of early apoptosis, in comparison with those in the control cells. In the adenocarcinoma cells, the percentage of apoptotic cells increased 24–48 h after transfection (Figure 5A, *p* < 0.01). This could be caused by the activation of the compensation mechanism, of which EpCAM is included. On the other hand, the percentage of apoptotic cells was increased in all of the investigated cell lines (Figure 5D,E) after the KO of lncCDH5-3:3.

**Figure 6:**
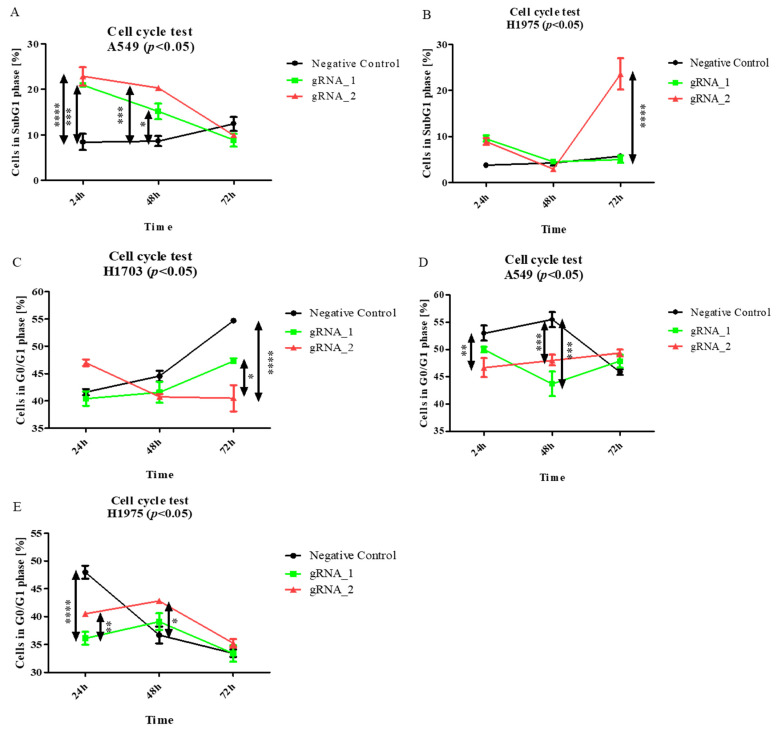
The effects of lncCDH5-3:3 silencing on the apoptosis intensity (cells in SubG1 gate) (**A**,**B**), and cell cycles (**C**–**E**) of cultured cells: A549, H1975, and H1703 cells. The cell cycle study was carried out by cell cycle assay. Analysis was performed at 24, 48, and 72 h post transient transfection. Data are presented as median values and minimum (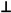) and maximum (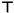) values from three biological replicates (*n* = 3). The *p*-values were calculated on the basis of the Friedman ANOVA and Dunn’s comparative post hoc tests. Statistically significant results are marked: * *p* < 0.05, ** *p* < 0.01, *** *p* < 0.001, and **** *p* < 0.0001. Medians are marked as circles, squares, and triangles; 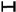 represents the *p*-value from post hoc test.

LncCDH5-3:3 knockout affects apoptosis in adenocarcinoma cells, and it is associated with an increased percentage of cells in the SubG1 gate. Particularly, as is shown in Figure 6A, the largest percentages of A549 cells in the SubG1 gate were detected 24 h and 48 h after transfection (*p* < 0.0001 and *p* < 0.001, respectively), compared to the control. Moreover, the lncCDH5-3:3 knockout significantly increased (*p* < 0.0001) the percentage of H1975 cells in this gate 48 h post transient transfection (Figure 6B). However, no significant change in the H1703 cell percentages in the SubG1 population was observed (data not shown).

The obtained results suggest that the silencing of lncCDH5-3:3 expression probably leads to cell death in adenocarcinoma cell lines. However, lung cancer cell lines with lncCDH5-3:3 knockout showed no decrease in the percentages of cells in the G0/G1 phase (Figure 6C–E). Such an effect may be associated with the statistically higher percentage of apoptotic cells in the SubG1 phase compared to the control cells, as was shown before (Figure 6A,B).

**Figure 7:**
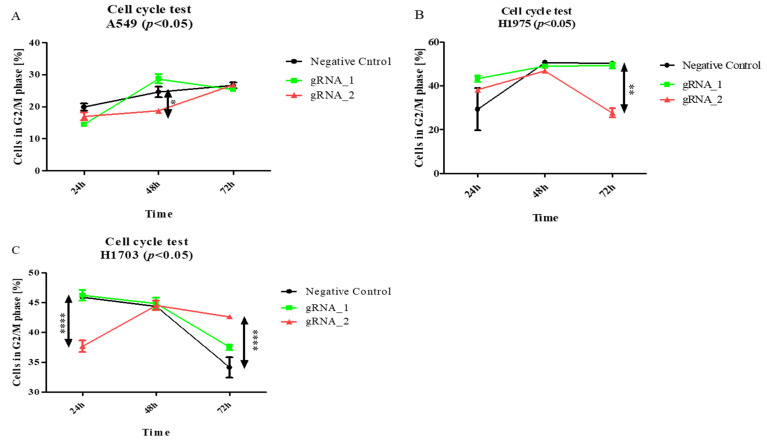
The effects of lncCDH5-3:3 silencing on the distribution of cultured cells: A549 (**A**), H1975 (**B**), and H1703 cells (**C**) in the G2/M phase of the cell cycle. The cell cycle study was carried out by cell cycle assay. Analysis was performed at 24, 48, and 72 h post transient transfection. Data are presented as medians from three biological replicates. The *p*-values were calculated on the basis of the Friedman ANOVA and Dunn’s comparative post hoc tests. Statistically significant results are marked as * *p* < 0.05, ** *p* < 0.01, and **** *p* < 0.0005. In this figure, the maximum values are marked as 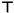; the minimum values as 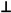; the medians as circles, squares, and triangles; and 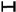 represents the *p*-value from the post hoc test.

Then, to examine the effect of the lncCDH5-3:3 knockout on other phases of the cell cycle in the studied lung cancer cell lines, the above assay was applied. As is shown in Figure 7A, the percentage of A549 cells in the G2/M phase significantly decreased (*p* < 0.05) 24 h after transfection, and, after 48 h, it returned to the level of the control cells. On the other hand, this is a percentage of the decrease (*p* < 0.01) in the H1975 cells in the G2/M phase, 48 h post transient transfection, compared to the control cells (Figure 7B). In turn, Figure 7C demonstrates, in the H1703 squamous cell carcinoma line, 24 h after transfection, the increased percentage of cells in the G2/M phase (*p* < 0.05).

Hence, the above data suggest that lncCDH5-3:3 likely regulates signaling pathways that promote proliferation or S-to-G2/M phase transition in adenocarcinoma cells.

### 3.4. Effects of LncCDH5-3:3 Overexpression on Cell Cycle, Cell Proliferation, Cell Migration, and Apoptosis in Lung Cancer Cells

To confirm our hypotheses, we performed a study of the influence of lncCDH5-3:3 overexpression in lung cancer cells on the cell cycle and apoptosis, as well as on the expression levels of proteins associated with cell proliferation, migration, or the EMT. For this, NSCLC cell lines were transfected with pcDNA3.1 plasmid encoding lncCDH5-3:3, whereas cells transfected with empty pcDNA3.1 were used as a control. To study the effect of lncCDH5-3:3 overexpression on apoptosis and the cell cycle, Annexin V staining and cell cycle assays were performed.

**Figure 8:**
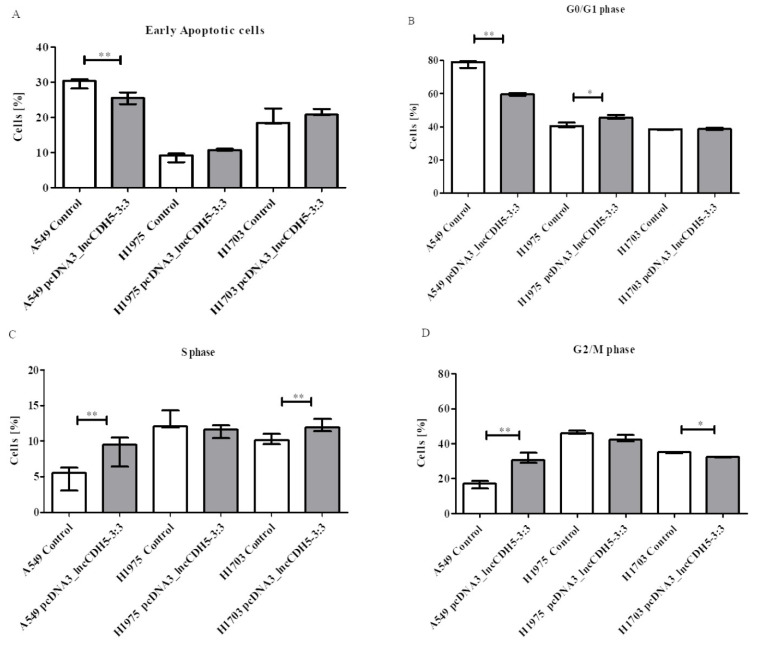
The effects of lncCDH5-3:3 overexpression on the percentages of early apoptotic cells (**A**), or cells in the G0/G1 (**B**), S (**C**), and G2/M (**D**) cell cycle phases of the cultured cells of the A549, H1975, and H1703 cell lines. The effect of lncCDH5-3:3 silencing on the apoptosis intensity was carried out by Annexin V staining, while the cell cycle study was carried out by cell cycle assay. Data are presented as median values and minimum (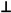) and maximum (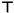) values from three biological replicates (*n* = 3). The *p*-values were calculated on the basis of the Wilcoxon test. Statistically significant results are marked as * *p* < 0.05 and ** *p* < 0.01; 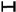 represents the *p*-value from the post hoc test.

As is shown in Figure 8A, lncCDH5-3:3 overexpression lowers (*p* < 0.01) the percentage of early apoptotic cells of the A549 line, in comparison to the control cells. Therewith, a statistically significant decrease (*p* < 0.01) in the percentage of these cells in the G0/G1 phase was observed, although the percentage of H1975 cells in this phase of the cell cycle was significantly increased (*p* < 0.05) (Figure 8B). Moreover, Figure 8C illustrates increases in the percentages of A549 and H1703 cells (*p* < 0.05 and *p* < 0.01, respectively) in the S phase of the cell cycle. A statistically significant decrease (*p* < 0.01) was also demonstrated in the percentage of A549 cells in the G2/M phase, while the percentage of H1703 cells was significantly lower (*p* < 0.05) after lncCDH5-3:3 overexpression (Figure 8D). However, the percentages of H1975 cells in the S and G2/M phases, as well as the percentage of early apoptotic H1975 cells, were insignificant in comparison to the control cells (Figure 8A,C,D). As is shown in Figure 8A,B, no statistically significant differences were found in the percentage of cells in the G0/G1 phase of the cell cycle, nor in the early apoptotic cells of the H1703 line.

In conclusion, the number of H1975 and H1703 cells in the S phase of the cell cycle is either unaffected or slightly increased by lncCDH5-3:3 overexpression, whereas the number of A549 cells in the S phase is increased by 1.54 ± 0.47 folds, and in the G2/M phase by 1.87 ± 0.16 folds. These data indicate that lncCDH5-3:3 exerts a remarkable stimulatory effect on A549-cell proliferation.

**Figure 9:**
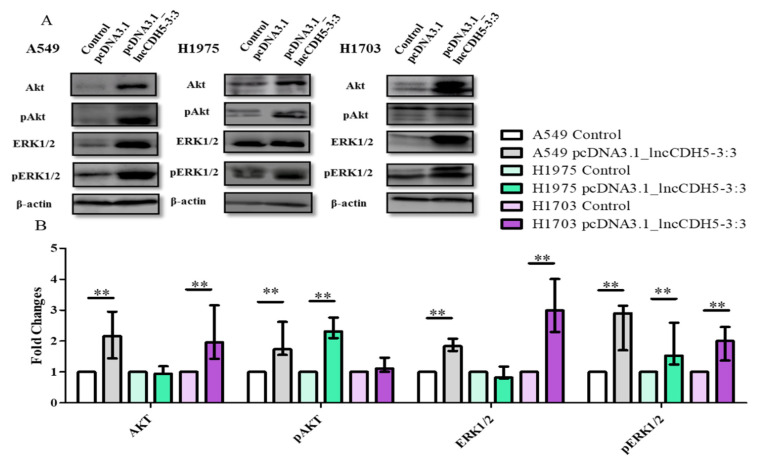
Western blot images (**A**), and fold changes (**B**) of Akt, pAkt, ERK1/2, and pERK1/2 expressions in response to lncCDH5-3:3 overexpression in cultured A549, H1975, and H1703 cells. In Western blot assay, β-actin was used as a loading control. The bar graph represents the median fold change and the minimum (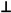) and maximum (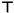) values of three independent replicates, and data are presented as fold changes relative to the control. The *p*-values were calculated on the basis of the Wilcoxon test. Statistically significant results are marked as ** *p* < 0.01; 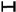 represents the *p*-value from the post hoc test.

Two major cell survival pathways that are upregulated and activated in lung cancer tissues are mediated by Akt and ERK1/2, which are known to play a crucial role in the regulation of cellular proliferation, differentiation, and migration [22]. Hence, we performed a study of the possible role of lncCDH5-3:3 in the regulation of lung cancer cell proliferation via the Akt- and ERK1/2-mediated signaling pathways. For this purpose, the expression levels of total Akt and ERK1/2, as well as their phosphorylated forms (pAkt and pERK1/2, respectively), were evaluated in cells overexpressing lncCDH5-3:3 using Western blot analysis (Figure 9A). As is shown in Figure 9B, the lncCDH5-3:3 overexpression causes different effects in the three lung cancer cell lines: in A549 cells, it increases the levels of Akt, pAkt, ERK, and pERK; in H1975 cells, it increases the levels of pAkt, and pERK; and in H1703, it increases the levels of ERK and pERK. On the other hand, the adenocarcinoma cell lines showed an increased level of pAkt in response to lncCDH5-3:3 overexpression. Additionally, the three cell lines showed the enhancement of ERK phosphorylation during lncCDH5-3:3 overexpression.

An analysis of the percentages of cells in the S and/or G2/M phases, and the expressions of proteins involved in proliferation, suggests that a higher expression of Akt may cause increased levels of cells in the S and/or G2/M phases.

The differences in the results obtained after overexpression could be because of the type of tumor (adenocarcinoma or squamous cell carcinoma), or the differences in the occurring mutations that are present in the investigated cell lines. These cell lines were chosen because of their gene mutations, which are the most frequently occurring in NSCLC patients, e.g., A549: mutations in the *KRAS* gene; H1975: mutations in the *EGFR* gene; and H1703; mutations in the *Tp53* gene. Nonreproducible results between the different cell lines connected with proliferation, pluripotency, and apoptosis were expected because of the abovementioned mutations and as a consequence of the different compensation mechanisms of cancer cells. We think that the fact that the influence of lncCDH5-3:3 on the functioning of NSCLC cells depends on the mutation characteristics for cancer cells is also a useful observation for future research. In future studies, the mutational status of cells should also be considered.

**Figure 10:**
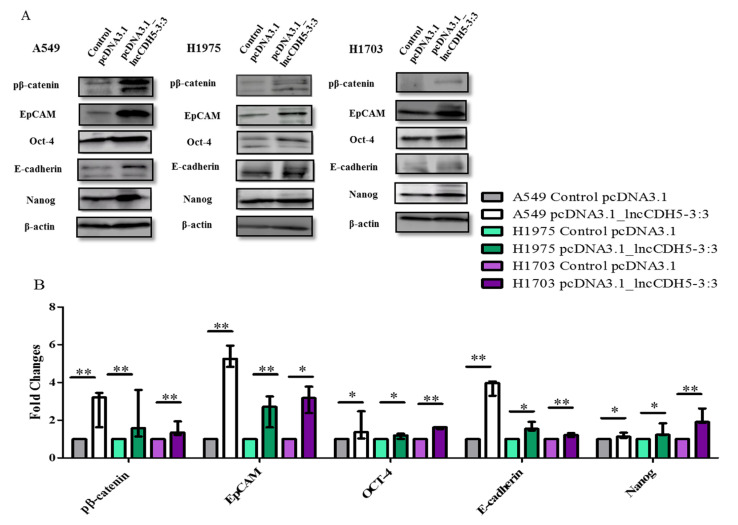
Western blot images (**A**), and fold changes (**B**) of pβ-catenin, Oct-4, EpCAM, E-cadherin, and Nanog expressions in response to lncCDH5-3:3 overexpression in cultured A549, H1975, and H1703 cells. β-actin was used as a loading control in the Western blot assay. The bar graph represents the median and the minimum (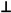) and maximum (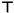) values of three independent replicates, and the data are presented as fold changes relative to the control. The *p*-values were calculated on the basis of the Wilcoxon test. Statistically significant results are marked as * *p* < 0.05 and ** *p* < 0.01; 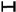 represents the *p*-value from the post hoc test.

We also studied the outcome of lncCDH5-3:3 overexpression in lung cancer cell lines on the expression of EMT-associated proteins, such as E-cadherin and EpCAM. Figure 10A,B demonstrates the influence of lncCDH5-3:3 overexpression on the expression of the above proteins in NSCLC cell lines. In particular, all of the studied proteins showed significantly increased fold changes in their expression levels, in comparison to the control cells (Figure 10B). Additionally, lncCDH5-3:3 overexpression significantly increased (*p* < 0.05 or *p* < 0.01, depending on the cell line) the expressions of transcription factors, such as Oct-4 and Nanog.

It is known that EpCAM, E-cadherin, Oct-4, and Nanog expressions are regulated by β-catenin, which is present in the cell nucleus [23,24]. However, we show that β-catenin is probably not required for these protein expressions in lung cancer cells. As is represented in Figure 10B, the lncCDH5-3:3 overexpression significantly increased (*p* < 0.05 for A549 and H1975 cells, *p* < 0.01 for H1703 cells) the levels of phosphorylated β-catenin, which indicates its increased degradation. This suggests that lncCDH5-3:3 may regulate EpCAM and E-cadherin expressions, independently of β-catenin.

It can be concluded that the overexpression of lncCDH5-3:3 likely leads to the activation of compensatory mechanisms, which promote the expressions of the proteins involved in cell proliferation (Akt, ERK1/2, Ep-CAM, Oct-4, and Nanog). It is likely that the expressions of these proteins are not β-catenin-dependent. Moreover, along with lncCDH5-3:3 overexpression, increased E-cadherin levels were also detected, which may indicate the decreased invasive capacity of the cancer cells.

Hence, lncCDH5-3:3 plays an important role in cancer cell biology by regulating apoptosis and proliferation. The time at which cells compensate for lncCDH5-3:3 silencing by other proliferation- and/or apoptosis-associated proteins is dependent on the cell line. The mutation that causes epidermal growth factor receptor (EGFR) activation (L858R, in the case of the H1975 cell line) is not sufficient to compensate for the caspase-3/7-induced apoptotic pathway. However, the lncRNA expression must be balanced and strictly controlled in cancer cells. As previously mentioned, lncCDH5-3:3 overexpression leads to the activation of Akt- and ERK1/2-associated signaling pathways in lung cancer cells. On the other hand, Wnt/β-catenin signaling is inactivated because of the high level of phosphorylated β-catenin that indicates its degradation. The in vitro studies show that lung cancer cells lose their invasive abilities by increasing the E-cadherin expression and activating proliferative pathways. Moreover, the highest lncCDH5-3:3 expression level was indicated in stage III NSCLC. It is shown that NSCLC cells in this stage demonstrate a low migration ability and the highest proliferative activity, which causes tumor growth. Such a variant of lncCDH5-3:3 expression regulation is also presented in the adenocarcinoma cells of A549 and H1975 lines after the failed lncCDH5-3:3 knockout. The cancer cells expressed lncCDH5-3:3 for a short time to enhance proliferation. Thereafter, the level of lncCDH5-3:3 expression decreased, possibly in order to increase the migration capacity of cells.

## 4. Discussion

The molecular mechanisms that define the aggressiveness of malignant lung tumors, and their low responsiveness to anticancer treatments, are poorly understood. In our investigation, we tried to validate the potential role of lncCDH5-3:3 as an oncogene. In the in silico analysis of the lncCDH5-3:3 RNA stem–loop structure we performed, it revealed its very stable secondary structure (ΔG = −241.38 kcal/mol). This might explain the success in lncCDH5-3:3 detection, but an additional study is required for the proper investigation of its life span. The presence of long, and therefore stable, stem structures in the lncCDH5-3:3 transcript could also potentially explain the fact that there is no protein-coding potential. We found two translation termination codons, at distances of 24 bp and 6 bp from the optimal Kozak sequences, which are at the positions of 132 and 255 (at the positions of 156 and 261, respectively) (Figure S1).

In our research, we focused on the lncCDH5-3:3-dependent changes in the expressions of genes and their protein products, which are involved in the regulation of cell growth and apoptosis, as well as in the migration and invasiveness of lung cancer cells. We show that the lncCDH5-3:3 knockout increased the percentages of apoptotic cells in both the lung adenocarcinoma and squamous cancer cell lines (Figure 5D–F). Therewith, decreases in the percentages of viable adenocarcinoma cells, in both the Annexin V staining and the caspase-3/7 assay, were demonstrated (Figure 4). Additionally, we show an increase in the percentage of apoptotic adenocarcinoma cells using the SubG1 method of cell death detection [25]. In particular, the percentage of A549 cells in the SubG1 gate increased after 24 h, while, for the H1975 cells, the increase was 48 h post transient transfection (Figure 6A,B). This effect of lncCDH5-3:3 silencing can be explained by the activation of various apoptosis-induced compensatory mechanisms, which are dependent on the cell line [26]. The decrease in the lncCDH5-3:3 expression was also accompanied by an increase in the activity of caspases 3 and 7. In the case of squamous cancer cells, especially, increases in the caspase-3 and capase-7 activations were observed 72 h after the lncCDH5-3:3 knockout (Figure 5F). Huang et al. also demonstrated an increased percentage of apoptotic cells in the SubG1 gate; however, it was in response to the lncRNA MEG3 overexpression in the esophageal squamous cell carcinoma lineage [27]. On the other hand, in vitro studies did not reveal an increase in the percentage of apoptotic cells in the SubG1 gate in response to MA-linc1 silencing in the osteosarcoma lineage [28].

Interestingly, as is shown in Figure S1, the CRISPR-Cas9-system-mediated lncCDH5-3:3 knockout led to the appearance of more than one DNA band (DNA ladder), as well as to their disappearance 48 h post transient transfection in the H1703 cells. Such an effect most likely indicates the apoptosis of these cells. Studies performed by Jänicke et al. demonstrate a caspase-3 activation that causes DNA fragmentation and that resulted in the appearance of a DNA ladder on the agarose gel [29]. These results are consistent with the results we obtained for the gRNA_1 and gRNA_3 pairs. It should be noted that the DNA bands obtained in the adenocarcinoma lines 48 h after transfection also lose their sharpness, which suggests apoptosis due to caspases-3 and capase-7 activation (Figure S1). To the best of our knowledge, we cannot compare the research data for the lncCDH5-3:3 functions obtained in our study with the results of the other authors because of the lack of articles that have demonstrated the role of lncCDH5-3:3 in lung cancer cells. Thus, we decided to compare the obtained results with those presented by other researchers that have studied different lncRNAs. Thus, Liu and Guiting´s research shows that lncRNA BANCR overexpression also activated caspases 3 and 7 through the regulation of Bcl-2 expression in NSCLC cells. Moreover, they demonstrated, as well as we did (Figure 5), that the activation of caspases 3 and 7 occurs 72 h post transient transfection [30]. Another lncRNA, named “lncRNA ANRIL”, which is similar to lncCDH5-3:3, affects apoptosis in lung cancer cells. In particular, lncRNA ANRIL inhibits programmed cell death via miR-99a and miR-449a silencing [31]. It also shows a higher expression level in tumors than in healthy tissues [21]. In turn, lncRNA MEG3 regulates apoptosis through p53 activation [30]. The overexpression of lncRNA PANDAR induced apoptosis in NSCLC cells. Moreover, this lncRNA showed lower expression levels in tumors than in normal tissue. Moreover, increased lncRNA PANDAR expression has been observed in disease stages I and II, compared to stages III and IV [32]. LncRNA MEG3 expression in NSCLC patients, such as lncCDH5-3:3, correlates with the disease severity. However, unlike lncCDH5-3:3, its expression is higher in disease stages I and II compared to stages III and IV [33]. The expression of lncRNA ANRIL, as well as of lncCDH5-3:3, are higher in disease stage III than in stages I and II [34]. As is shown in other studies, lncRNA expression also correlates with the NSCLC type. For example, lncCCAT2 demonstrated higher expression levels in adenocarcinoma cells than in squamous cell carcinoma cells [35]. Instead, in our research, lncCDH5-3:3 did not show significant differences in its expression levels depending on the tumor type.

**Figure 11:**
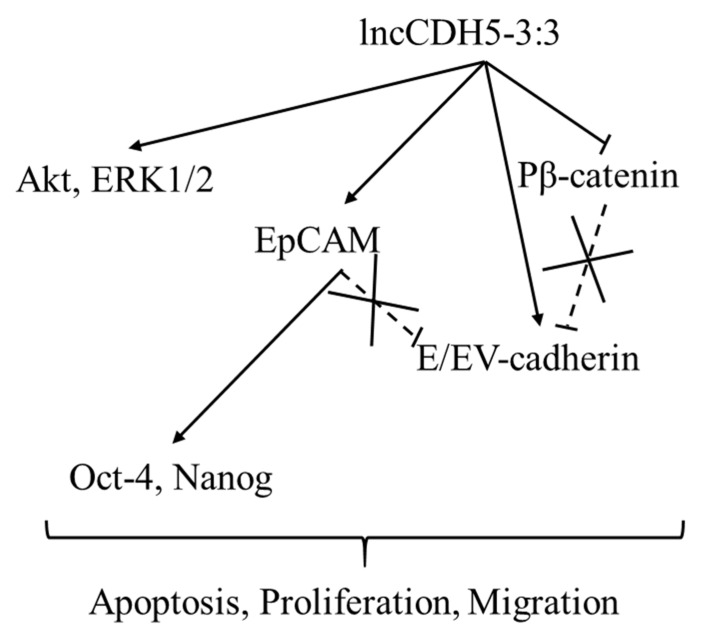
The role of lncCDH5-3:3 in the cancer cells. The sharp arrowheads indicate the positive regulation of genes by lncCDH5-3:3, while the flat heads indicate the negative regulation, which causes the degradation of β-catenin. The crossed flathead arrows show the lack of protein interactions reported in the literature.

In the current study, we demonstrate that lncCDH5-3:3 silencing caused the accumulation of H1975 cells in the G0/G1 phase of the cell cycle, 48 h post transient transfection. However, the percentages of A549, H1975, and H1703 cells in the G2/M phase were decreased. In the case of the H1975 line, it was observed 48 h post transient transfection, while, in the A549 cells, it was observed 24 h post transient transfection (Figure 7A,B). The response to lncCDH5-3:3 silencing may likely be cell-line-specific because of the present mutations, and, at the same time, dependent on the activation of compensatory pathways. Thus, lncCDH5-3:3-mediated decreases in the percentages of cells in the G2/M phase of the cell cycle, and increases in the G0/G1 phase, were demonstrated in the NSCLC cell lines. Such effects are probably caused by the reduced expressions or activity of cell-proliferation-related proteins, such as ERK1/2 and Akt. Additionally, changes in the expression regulation of EpCAM, as well as in the EpCAM-associated transcription factors (Oct-4 and Nanog), can also play a role (Figure 6) [23,36,37]. In particular, the overexpression of lncCDH5-3:3 increased the expression level of EpCAM, as well as of Oct-4 and Nanog (Figure 10A,B). The elevated level of phosphorylated β-catenin, which could indicate its degradation, was also observed in the study and is probably proof of the Wnt signaling pathway switching off upon lncCDH5-3:3 overexpression in human lung cancer cells [36]. As it turns out, Oct-4 expression is not only β-catenin-dependent. Eberl et al. show that Oct-4, by itself, may also be a transcriptional activator for the *OCT-4* gene [38]. In NSCLC cell lines, the increased levels of phosphorylated β-catenin negatively correlate with the Nanog and E-cadherin expressions levels (Figure 10 and Figure 11).

On the other hand, Nanog activation occurs not only through the Wnt/β-catenin signaling pathway, but can also be promoted by the TGFβ/SMAD2/3 signaling pathway [39,40]. Jameelah et al. show that Oct-4 and Nanog, in different manners, regulate the lncRNAs expression in embryonic stem cells. In particular, lncRNA AK028326 is upregulated by Oct-4, while lncRNA AK141205 is downregulated by Nanog [41]. Additionally, HERVH transcripts can function as lncRNAs that interact with OCT4 to regulate the expression of neighboring genes [13]. We show that the regulation of lncCDH5-3:3 expression is not limited to only the abovementioned, EpCAM, Oct-4, and Nanog. It is likely also modulated by proliferation-related proteins, such as ERK1/2 and Akt (Figure 9).

In summary, lncCDH5-3:3 can be considered as an oncogene. The silencing of lncCDH5-3:3 in NSCLC cells promotes apoptosis via the extrinsic pathway, which activates caspases 3 and 7. In contrast, lncCDH5-3:3 overexpression stimulates the expressions of Akt and ERK1/2, or their active forms, which are involved in cell proliferation regulation and the EMT, depending on the cell line. On the other hand, lncCDH5-3:3 overexpression causes β-catenin degradation and the elevated expression of E-cadherin, which can inhibit the EMT process. Moreover, the low expression of E-cadherin may be associated with poor prognoses in lung cancer patients [42]. Additionally, Wang S. et al. show that high E-cadherin expression inhibits the migration of cancer cells. The high expression of E-cadherin was accompanied by lower expressions of MMP-9 and MMP-13 [43]. The lowest expression levels of lncCDH5-3:3 were determined in patients with stage II NSCLC, compared to those with stages I and III. Therefore, we propose that NSCLC cells maintain elevated lncCDH5-3:3 expression for a certain time during carcinogenesis, and in cancer cells with specific mutations, for example, in KRAS or p53, such as in A549 or H1703 cells, respectively. As was demonstrated, during lncCDH5-3:3 overexpression, balancing the ability to migrate and apoptosis can induce proliferation and block migration. This may result in tumor growth and the absence of the cancer cell migration ability, as was indicated for stage III of NSCLC. In addition, Warth et al. show that the highest expression levels of Ki67, which are a cellular marker of proliferation, were determined in NSCLC patients in stage III [44].

## 5. Conclusions

Our data demonstrate that lncCDH5-3:3 is an important molecule that orchestrates the malignancy of NSCLC via the promotion of the cell migration and invasion capabilities, likely by regulating the E-cadherin and/or EpCAM expressions. LncCDH5-3:3 silencing promotes apoptosis, as well as the expression of proteins that are responsible for cell proliferation.

## Figures and Tables

**Table 1:** gRNAs used for the lncCDH5-3:3 knockout.

Primer	Sequence (5′→3′)
gRNA_1F	CTGTAGTCCAGGAATAGTCA
gRNA_2R	TATTCTACACCTTGAAGGCG
gRNA_3R	TTTGTCTCACAGTGAAGGTA

## Data Availability

The data presented in this study are available upon request from the corresponding author. The data are not public because of the disclosure agreement.

## Supplementary Materials

The following supporting information can be downloaded at: https://www.mdpi.com/article/10.3390/cells11030378/s1, Table S1: Detailed characteristic of patients with NSCLC with stages I–III (adenocarcinoma: I-*n* = 3, II-*n* = 4, III-*n* = 3; squamous cell carcinoma I-*n* = 3, II-*n* = 3, III-*n* = 4); Figure S1: The sequence and structure of lncCDH5-3:3. (**A**) The lncCDH5-3:3 primary sequence and stop codons (marked in yellow), five AUG codons, at positions 132, 169, 255, 423, and 577 (marked in blue) are indicated. (**B**) Predicted optimal secondary structure consists of multiple stem–loop structures; Figure S2: Electrophoresis of cleavage products obtained after CRISPR-Cas9-mediated lncCDH5-3:3 knockout using the gRNAs presented in Table 1. The arrows show the PCR product (~200 bp) after lncCDH5-3:3 silencing; Figure S3: Expression of lncCDH5-3:3. (**A**) Basal expressions of A549, H1975, and H1703 cells. (**B**) Changes in expression of lncCDH5-3:3 after stable overexpression of lncCDH5-3:3 gathered from transfection pols of NSCLC cells; Figure S4: Exemplary dot plots of the lncCDH5-3:3 silencing effect on caspase-3 and -7 activation in lung cancer cells: control (**A**), and knockout of the lncCDH5-3:3 with the first (**B**) and second (**C**) pairs of gRNAs; Figure S5: Exemplary dot plots of the lncCDH5-3:3 silencing effect on the viability of lung cancer cells: control (**A**), and knockout of the lncCDH5-3:3 with the first (**B**) and second (**C**) pairs of gRNAs; Figure S6: Exemplary dot plots of the lncCDH5-3:3 silencing effect on lung cancer cell cycle: control (**A**), and knockout of the lncCDH5-3:3 with the first (**B**) and second (**C**) pairs of gRNAs.
