# Supplementary material for: LncCDH5-3:3 Regulates Apoptosis, Proliferation, and Aggressiveness in Human Lung Cancer Cells
Source: Cells. 2022 Jan 23;11(3):378. doi: 10.3390/cells11030378 (PMC8834634; doi:10.3390/cells11030378)
Supplement: Supplementary file 1 [file cells-11-00378-s001.zip › cells-1484317-supplementary.pdf]

cells

MDPI

Supplementary Materials:

Supplementary Table S1. Detailed characteristics of patients with NSCLC with stages I-III (adenocarcinoma: I–n=3, II–n=4, III–n=3; squamous cell carcinoma I–n=3, II–n=3, III–n=4).

Adenocarcinoma			Squamous Cell Carcinoma		
**I**	**II**	**III**	**I**	**II**	**III**
**n=3**	**n=4**	**n=3**	**n=3**	**n=3**	**n=4**
T1bN 0M0–IA	T2bN0M0–IIA	T3N2M0–IIIA	T1aNoM0–IA	T2aN1M0–IIA	T1aN2M0–IIIA
T1bN1M0–IA	T3N1M0–IIB	T4N1M0–IIIA	T2aN0M0–IB	T3N0M0–IIB	T3N1M0–IIIA
T1bN 0M0–IA	T1aN1M0–IIA	T2bN2M0- IIIA	T2aN0M0–IB	T2bN0M0–IIA	T3N0M0–IIIA
	T2bN0M0–IIA				T3N2M0–IIIA

A

lncCDH5-3:3

```
AGCCUAAUAAGGGAACUGGGCAGGUGGGGAUAACUAAAAAGGAGUGCUUA   50
AAAGAGUAUUUUCUGAGUUGGCACUAGAGUUGGGGAGUUUUAAGAGGUUU  100
AGAAGCCUGGCCGUCAAUACCCACAACAGUUAUGGAGGCAAGGGAAACAG  150
GCCCUUGAAAAGAAGGUAAUGUGGAGUGGGUAGCCUCCAUAUUGAUUAAG  200
AAGGGGACGGGCUUACCUUUCACUGUGAGAGUUACCCAAAGCUCGGCGU   250
CGUGAUGGUCUAGGGGGCUUCCGAGGCGAUCGGGCAGUGUCAGUCUUCA  300
GCCGCUAAGCCGAGAAGAUCUGGGAAGGAGUCAGUCAGAGAGCCUUGGGC  350
CAGAGUUCCAGGGGCUCUGGAGUGGCUGCCAGGUGAGUUGAACAGUCCG   400
UUUUCAGUGGGGUCCCACACAGAUGGGACGCGGCUUAGGAGGAAUCCCGG  450
GCAGCGGGCAUUCCUUGGCCCAGUGGCCAGAUUUCCGGCACGUGUAGCAA  500
GCUCCUGUGGGAGGAGGUUCUGGAGGAACACCUGGCCGCUGCGGUUCAGG 550
CGUUUGGAAGUUCUCGUGUGCUGGAGAUGUGGCUGGGGUUUGUCUCACAG 600
UGAAG                                              605
```

AUG codons at the positions of 132, 169, 255, 423 and 577
UAA codons at the positions of 5, 8, 31, 35, 49, 91,165, 197 and 306
UAG codons at the positions of 75, 100, 181, 261, 436 and 495
UGA codons at the positions of 64, 156, 193, 225, 386, 391 and 601

B

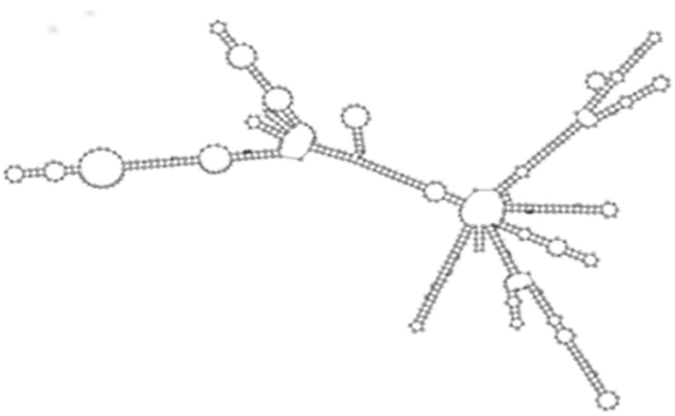

Supplementary Figure S1. The sequence and structure of lncCDH5-3:3. (**A**) The lncCDH5-3:3 primary sequence and stop codons (marked on yellow), five AUG codons at positions 132, 169, 255, 423, and 577 (marked on blue) are indicated. **(B)** Predicted optimal secondary structure consists of multiple stem-loop structures.

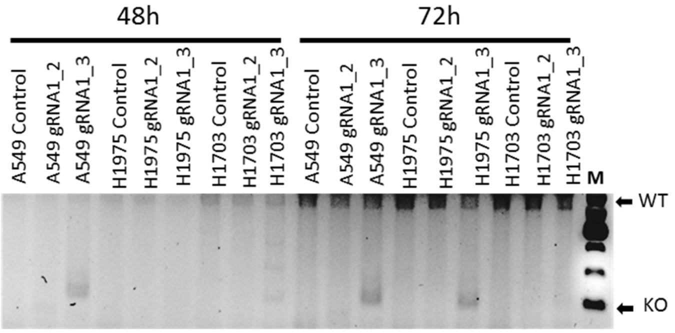

Supplementary Figure S2. Electrophoresis of cleavage products obtained after CRISPR-Cas9-mediated lncCDH5-3:3 knockout using gRNAs presented in Table 1. The arrows are showing PCR product (~200bp) after lncCDH5-3:3 silencing.

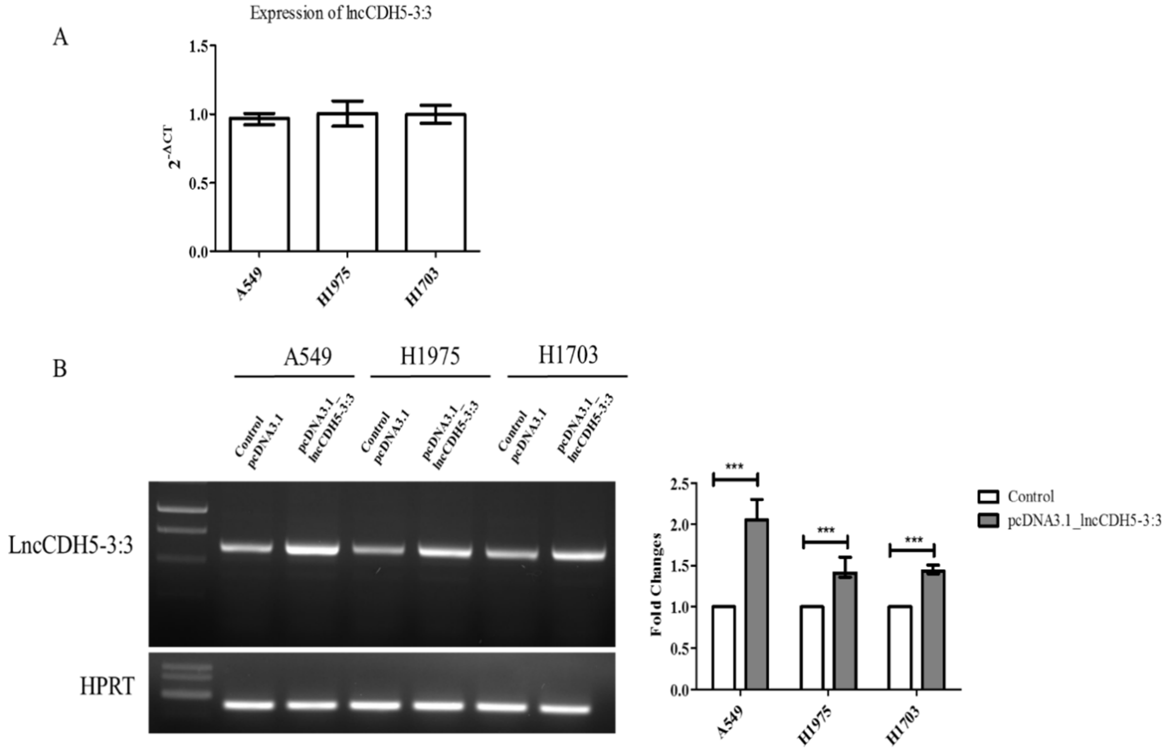

Supplementary Figure S3. Expression of lncCDH5-3:3. (**A**) Basal expression of A549, H1975, and H1703 cells. (**B**)Changes in expression of lncCDH5-3:3 after stable overexpression of lncCDH5-3:3 gathered from transfection pols of NSCLC cells.

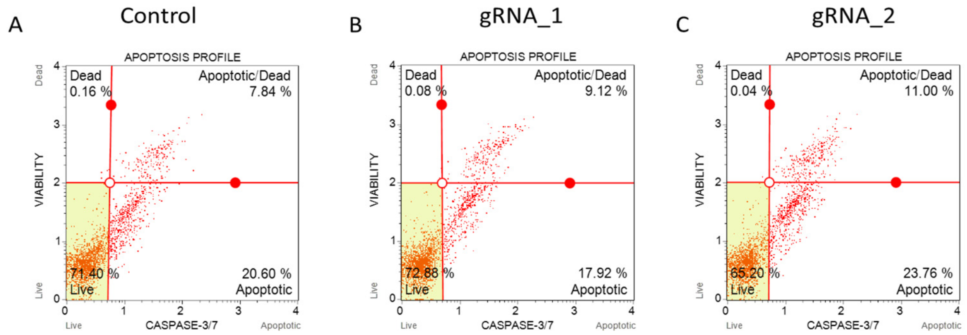

Supplementary Figure S4. Exemplary dot-plots of lncCDH5-3:3 silencing effect on caspase-3/7 activation in lung cancer cells: control (**A**) and knockout of the lncCDH5-3:3 with the first (**B**) and second (**C**) pair of gRNAs.

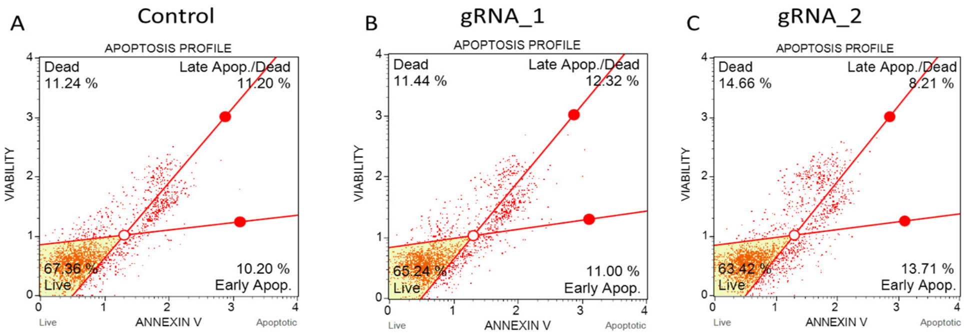

Supplementary Figure S5. Exemplary dot-plots of lncCDH5-3:3 silencing effect on the viability of lung cancer cells: control (**A**) and knockout of the lncCDH5-3:3 with the first (**B**) and second (**C**) pair of gRNAs.

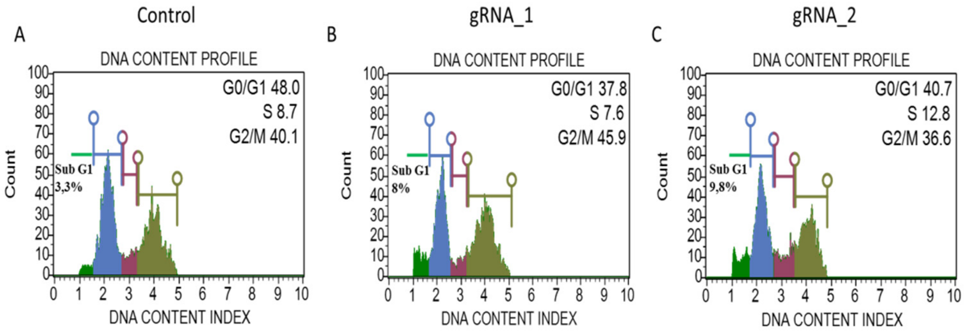

Supplementary Figure S6. Exemplary dot-plots of lncCDH5-3:3 silencing effect on lung cancer cell cycle: control (**A**) and knockout of the lncCDH5-3:3 with the first (**B**) and second (**C**) pair of gRNAs.
